# Global Systematic Review of the Measurement of Stigma Associated With People Living With Hepatitis B or Hepatitis C Viruses

**DOI:** 10.1111/jvh.70064

**Published:** 2025-08-26

**Authors:** Freddy Green, Ruth Simmons, David Leeman, Monica Desai, Matthew Hibbert

**Affiliations:** ^1^ Blood Safety, Hepatitis, Sexually Transmitted Infections and HIV UK Health Security Agency UK

**Keywords:** discrimination, hepatitis B virus, hepatitis C virus, hepatitis elimination, stigma, systematic review

## Abstract

Chronic hepatitis B and C affect over 300 million people globally. Despite treatment advances, stigma towards people living with hepatitis B/C (PLWHB/C) remains a barrier to care and impacts health outcomes. Addressing this stigma is key to achieving hepatitis elimination goals. This systematic review aims to synthesise existing approaches to measuring stigma experienced by PLWHB/C and examine factors associated with stigma in different social contexts. Databases searched included PubMed, PsycInfo and Web of Science, as well as grey literature (01/01/2008–30/06/2023). Studies were included if stigma experienced by or directed towards PLWHB/C was measured quantitatively. Data from included studies were synthesised using a narrative approach. Among 3053 studies, 81 were included. Various tools were used to measure internalised (e.g., self‐blame, shame), enacted (e.g., experiences of discrimination) and anticipated stigma (e.g., expectations of discrimination) related to PLWHB/C; most commonly the Toronto Chinese Hepatitis B Stigma Scale and Brener and Von Hippel's tool. Stigma was highly prevalent, impacting psychosocial wellbeing, treatment‐seeking behaviours and quality of life. Lower knowledge and conservative beliefs were linked to higher public stigma. Educational interventions and stigma‐reducing media showed some benefit in mitigating stigmatising attitudes. The review highlights stigma's pervasive nature and detrimental psychosocial impacts for PLWHB/C globally. While diverse measurement tools were used, standardising culturally validated instruments aligned with conceptual frameworks could improve research. Tailored educational initiatives could help reduce stigmatising attitudes. Crucially, stigma hindered timely diagnosis and treatment access, emphasising the need for multi‐level interventions addressing stigma to achieve elimination goals.

## 1 Introduction

Chronic viral hepatitis B (HBV) and hepatitis C (HCV) represent a significant global health concern with more than 250 million people and 50 million living with chronic HBV and chronic HCV respectively around the world [1, 2]. If left untreated, viral hepatitis can lead to high mortality rates resulting from complications such as cirrhosis and hepatocellular carcinoma [3]. In 2016, the World Health Organisation (WHO) set targets to eliminate viral hepatitis as a major public health threat by 2030 [4]. As with other elimination efforts, the hepatitis response requires an enabling environment of laws and regulations that support evidence‐based policies, promote and protect human and health rights and reduce stigma to ensure health equity.

Despite advances in prevention strategies and treatment regimens, people living with HBV or HCV (PLWHB/C) continue to face stigma and discrimination, impacting healthcare access, uptake and outcomes [5, 6]. There is evidence that stigma experienced by PLWHB/C can impact the patient pathway [7, 8, 9], as a barrier to testing [10, 11] and initiating treatment [12, 13]. In addition, experiencing hepatitis‐related stigma is associated with negative feelings and lower health‐related quality of life [14]. Thus, addressing stigma is a key step towards reaching national and global elimination targets, with countries required by WHO to end policies and practices that condone or encourage stigma against people living with hepatitis [4].

Understanding the nature and extent of stigma associated with HBV and HCV requires examining its various dimensions and exploring its associations with a range of factors, including demographic characteristics, clinical status and psychosocial variables [15, 16]. It is also crucial for developing and evaluating the impact of effective interventions and policies aimed at mitigating its adverse effects. However, the measurement of stigma is complex and multifaceted, requiring a comprehensive approach that captures its various dimensions across diverse populations and cultural contexts [17].

The primary aim of this global systematic review is to assess existing approaches to the measurement of stigma experienced by or directed to PLWHB/C. The secondary aims are to assess various associations with stigma examined in the literature, identify gaps in knowledge and inform future research and intervention efforts. By examining a diverse range of factors associated with stigma and evaluating the methodologies used to measure it, this review aims to improve the understanding of the complex interplay between stigma and HBV/HCV and offer insights into effective strategies for stigma reduction and health promotion.

## 2 Methods

### 2.1 Protocol and Registration

This systematic review was conducted based on the Preferred Reporting Items for Systematic Review and Meta‐Analysis (PRISMA) statement guidelines [18]. The protocol for the review was prospectively registered on the International Prospective Register of Systematic Reviews (PROSPERO, ID:CRD42023450277).

### 2.2 Search Strategy

In July 2023, the systematic literature search was conducted using three databases: PubMed, PsycInfo and Web of Science. These databases were selected based on other systematic reviews on similar topics related to blood borne virus stigmatisation [7, 11]. The search terms used to identify articles were (‘hepatitis’ OR ‘hcv’ OR ‘hbv’) AND (stigma OR discrim*). An attempt to find relevant grey literature, such as organisational reports, was undertaken through known community organisations (e.g., the World Hepatitis Alliance), web searches and public health organisations (e.g., The UK Health Security Agency, Centre for Disease Control and Prevention, Public Health Agency of Canada).

### 2.3 Eligibility Criteria

To meet the eligibility criteria, studies must have investigated stigma towards people living with HBV or HCV, or stigma experienced by people living with HBV or HCV and its impact. Eligible studies were required to be published between 1 January 2008 and 30 June 2023 and feature quantitative measures of stigma and/or discrimination. The start date of 1 January 2008 was selected to align with a paper investigating HIV stigma frameworks [19], which built on the first People Living with HIV Stigma Index published in 2008. Studies were excluded when they were not published in English and a translation could not be found, investigated non‐viral hepatitis or non‐hepatitis B or C, or only qualitative methods were used.

### 2.4 Study Selection

All studies reviewed through the electronic databases were collected and uploaded into Endnote version 21.2. Four stages were used to identify studies: identification, screening, eligibility and inclusion [18]. Each stage was carried out independently by two reviewers (FG and MH.). Any disagreements that arose on study inclusion between reviewers were resolved through discussion mediated by an additional reviewer (DL).

### 2.5 Data Extraction and Appraisal

Two reviewers (FG. and MH.) collected data from all studies independently. An Excel spreadsheet was used for data extraction with predetermined fields (PLWHB/C Data S1), which were agreed upon by the two reviewers (FG. and MH.).

The appraisal tool for cross‐sectional studies (AXIS [20]) was used to assess the quality of peer‐reviewed studies included in the review that were of cross‐sectional design. The AXIS tool has the benefit of providing the user the opportunity to assess each individual aspect of study design to give an overall assessment of the quality of the study. For non‐cross‐sectional studies, the Risk Of Bias In Non‐randomised Studies—of Interventions tool (ROBINS‐I [21]), which evaluates the risk of bias in estimates of comparative effectiveness (harm or benefit) of interventions from studies that did not use randomisation to allocate units (individuals or clusters of individuals) to comparison groups, was employed. Both reviewers (FG. and MH.) completed the quality appraisal for all studies. Any disagreements that arose in the appraisal of studies between reviewers were resolved through discussion mediated by an additional reviewer (DL.).

Due to the heterogeneity in the way stigma was measured and the outcomes relating to stigma, meta‐analyses could not be conducted. Therefore, a narrative synthesis approach towards the evidence was undertaken and results were reported following Synthesis Without Meta‐Analysis (SWiM) guidelines [22].

The type of stigma examined for each study was grouped into three categories: internalised, enacted and anticipated stigma [17]. Internalised stigma refers to endorsing negative feelings and beliefs associated with hepatitis and applying them to the self. Enacted stigma involves experiences of discrimination, stereotyping and/or prejudice from others in the past or present due to an individual's hepatitis status. Finally, anticipated stigma involves expectations of discrimination, stereotyping and/or prejudice from others in the past or present due to an individual's hepatitis status.

The study population was grouped into three categories: people living with hepatitis, people not living with hepatitis, or both. Some studies aimed to recruit members of the public but did not ask whether participants were PLWHB/C. These studies were grouped as people not living with hepatitis to explore their attitudes towards PLWHB/C.

## 3 Results

**FIGURE 1:**
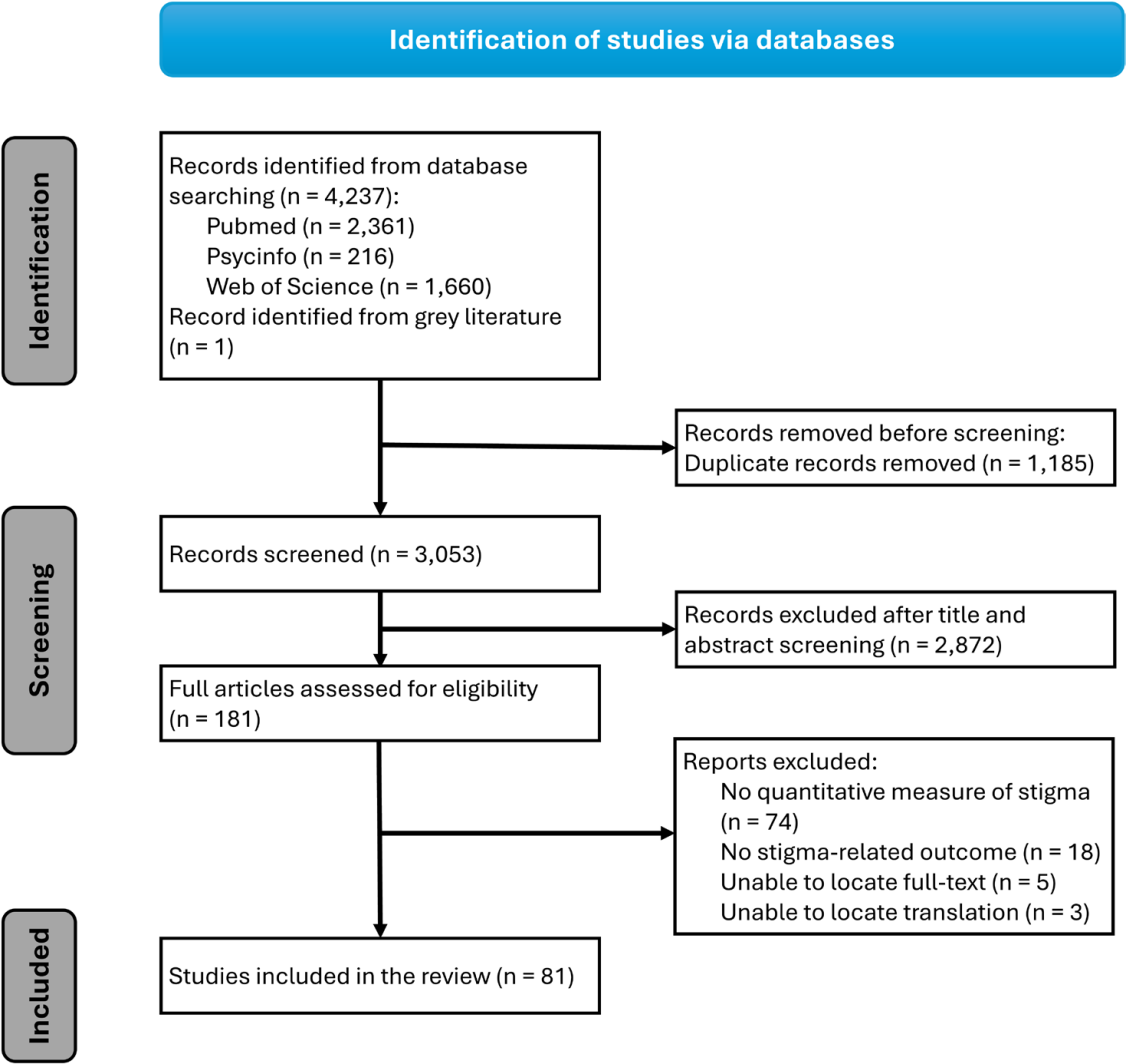
PRISMA flow diagram for article selection for systematic review of the measurement of and associations with stigma experienced by people living with HBV or HCV.

The search yielded 3053 studies. During title and abstract screening, 2872 studies were excluded as they did not meet the eligibility criteria. Assessment against eligibility criteria at the full text review stage (*n* = 181) excluded a further 100 studies. A total of 81 studies were reviewed in full (Figure 1).

A table summarising studies included in our systematic review of HBV and/or HCV stigma is presented in Data S2. All studies were deemed at an acceptable threshold for inclusion after applying the AXIS tool for cross‐sectional studies and ROBINS‐I for non‐cross‐sectional studies by both reviewers.

**FIGURE 2:**
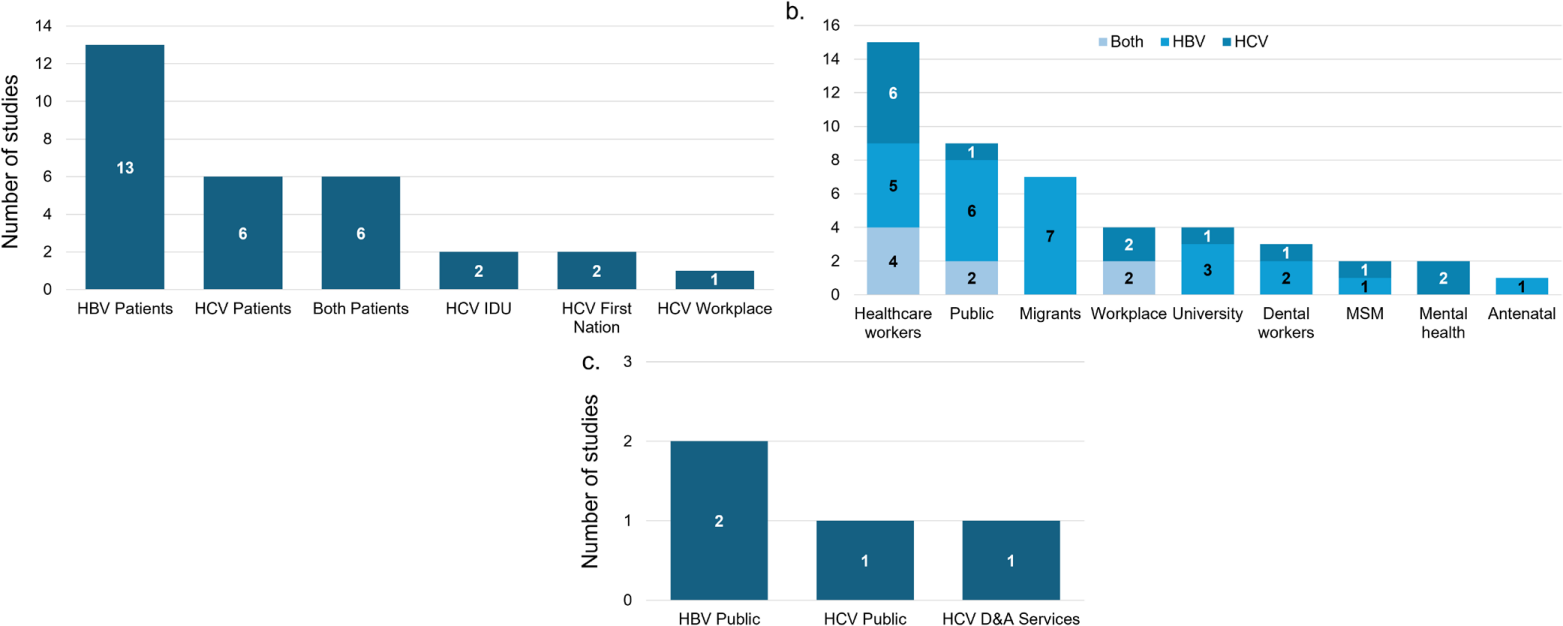
Population studied among studies focused on (a) individuals living with hepatitis, (b) individuals not living with hepatitis* and (c) individuals living with hepatitis and individuals not living with hepatitis*. *Studies that did not explicitly state whether PLWHB/C were recruited were grouped as not known to be living with hepatitis.

Of the 81 studies included, 30 had a study population of people living with hepatitis (37%), 47 on people not living with hepatitis (58%) and 4 on both populations (5%). This equated to 66,213 participants not living with hepatitis included in the review, 5615 participants living with hepatitis B and 2975 participants living with hepatitis C included. In terms of hepatitis virus focus, 40 studies (50%) focused on HBV, 27 (33%) focused on HCV and 14 (17%) included both. Further details of the population group included in each study can be seen in Figure 2.

**FIGURE 3:**
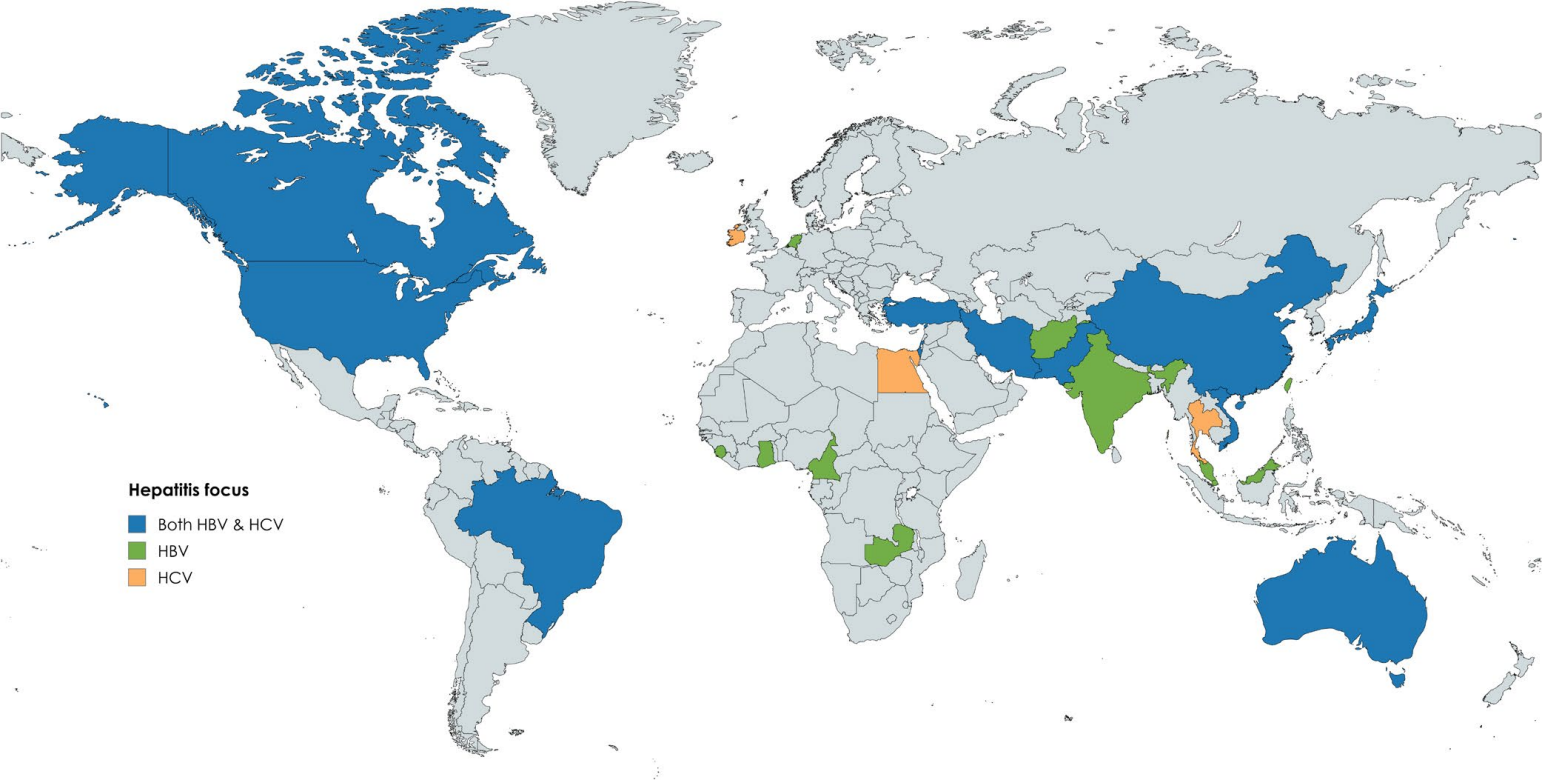
Geographical representation of countries that contributed studies to the review by hepatitis focus.

In terms of geographic spread of study country of focus (Figure 3), 37 were from Asia (46%) with China in particular contributing 13 (16%). Australia was the most common country of focus, contributing 15 studies (19%). North America and Africa contributed 14 and 6 studies, respectively (18% and 8%). Finally, Europe and South America contributed the fewest studies, with 5 and 3, respectively (6% and 4%). Of the 24 countries that had a study included in the review, 50% had only one study from that country.

### 3.1 Measurement of Stigma

A variety of adapted tools were used to measure stigma. There were two tools used frequently in the studies included in the review. The first was the Toronto Chinese Hepatitis B Stigma Scale (developed by Li et al., 2012 [23]). This tool was used to assess both internalised and enacted stigma associated with HBV and was originally used among Chinese people living in Canada who were not living with hepatitis. It was utilised in nine studies [23, 24, 25, 26, 27, 28, 29, 30, 31] mostly recruiting Asian people, with four studies taking place in China. The second was a tool developed by Brener and Von Hippel (2008) [32]. This tool was also used to assess both internalised and enacted stigma and was originally developed using psychology students. Brener & Von Hippel (2008) found that stigmatising attitudes towards PLWHC were also associated with other stigmatising attitudes (towards people living with HIV, men who have sex with men [MSM], people who inject drugs [PWID]), as well as religious fundamentalism and conservatism [32]. All nine studies adapting Brener & Von Hippel (2008) in this review were conducted in Australia, mostly among healthcare workers (*n* = 4), but the tool was also utilised for MSM and First Nation people, as well as PWID [33, 34, 35, 36, 37, 38, 39, 40, 41]. Both of Li et al. (2012) and Brener & Von Hippel (2008) were used in studies which examined people living with hepatitis and people not living with hepatitis. Both tools also appeared to be used consistently across the systematic review study period (2008–2023) from when they were conceptualised. Other tools used included Ishimaru et al. (2016) [42] used in four studies [43, 44, 45, 46] which were utilised in both HBV and HCV contexts, and Richmond et al. (2007) [47] also utilised in four studies [48, 49, 50, 51] investigating stigma associated with HCV.

Table 1 presents details on tools utilised in more than one study among papers included in the review. Typically, commonly used survey instruments reported on above comprised of Likert Scale based questions, asking respondents for responses on statements such as ‘People with hepatitis B are unclean’ ranging from ‘Strongly agree’ to ‘Strongly disagree’. Internal reliability of Brener & Von Hippel (2008) (Cronbach's alpha = 0.69, [38, 40]) was adequate and internal reliability of the Toronto Chinese Hepatitis B Stigma Scale [23] was high (Cronbach's alpha = 0.90). No reliability testing for Ishimaru et al. (2016) or Richmond et al. (2007) could be found.

**TABLE 1:** Detail on tools in more than one study among papers included in the review.

Tool	Number of studies included in the review using tool	Description	Type of stigma studied	Uses	Initial validity/reliability measures	Strengths	Limitations
Toronto Chinese Hepatitis B Stigma Scale (Li et al., 2012) [23]	9	20 items derived and modified from stigma scales in HIV/AIDS literature. Each item consists of a statement pertaining to HBV stigma, such as ‘People with hepatitis B are unclean’. Participants asked to indicate their opinion on a five‐point Likert scale.	Internalised/Enacted/Anticipated	People living with hepatitis People not living with hepatitis HBV (adapted for HCV in two studies)	Cronbach's alpha for the 20‐item scales was 0.90, indicating high reliability (internal consistency) of the scale.	Has been applied internationally (North America, Asia and Africa) High internal reliability	Scale items are all negatively framed and may be more subject to acquiescence response bias
Brener & Von Hippel, 2008 [32]	9	9 items adapted from the preexisting validated scale, AIDS Attitude Scale (Shrum, Turner and Bruce, 1989). Each item looks to measure attitudes towards HCV, such as ‘Only disgusting people get hep C infection’. Participants asked to indicate their opinion on a five‐point Likert scale.	Internalised/Enacted	People living with hepatitis People not living with hepatitis HCV (adapted for HBV in one study) Adapted to key populations Healthcare workers	Reliabilities of derived scale similar to those of parent scale. Correlations between attitudes towards HCV and homosexuals/IDUs significant.	Has been tailored to different key populations (men who have sex with men, people who inject drugs) Short scale and therefore easy to administer High construct validity	All studies in this review using this scale were from Australia, and therefore does not appear to be used more widely
Ishimaru et al., 2016 [42]	5	Questionnaire developed by authors specifically for study. Attitudes towards colleagues living with hepatitis assessed by four items relating to (a) willingness in accepting colleagues, (c) avoiding contact, (c) anxiety regarding potential infection and (d) assumption that a person living with hepatitis may be homosexual, have multiple sexual partners or be a drug user. Participants asked to indicate their opinion on a five‐point Likert scale.	Internalised/Enacted	Healthcare workers HBV and HCV	No validity or reliability statistics could be found for this tool.	Short tool and therefore easily applied in a healthcare context Used largely in Asia but could be applied to healthcare settings worldwide	Initial tool measures HBV and HCV stigma together, although levels of stigma and discrimination may be slightly different due to common routes of acquisition An understanding of the tool's reliability and validity is needed
Richmond et al., 2007 [47]	4	Questionnaires were specifically designed for the study as authors stated no instrument was located that explored health professional attitudes towards treating people with hepatitis C in the detail required. Tool assesses knowledge as well as attitude towards people living with hepatitis. The tool was implemented among healthcare professionals	Internalised/Enacted	Healthcare workers HCV only	No validity or reliability statistics could be found for this tool.	Has been applied internationally	A long scale to be administered in a healthcare context Would have benefited from factor analysis in its design
Cotler et al., 2012 [5]	2	Instrument consists of questions on demographics, HBV knowledge and stigma. Items organised into five domains and designed to assess perceptions of HBV stigma in the general population. Domains included (a) ‘Negative perception’, (b) ‘Social isolation’, (c) ‘Fear of contagion’, (d) Healthcare neglect and (e) workplace/school stigma	Internalised/Enacted	People living with hepatitis People not living with hepatitis HBV only	Cronbach's alpha for the full scale was 0.85 and high internal consistency between scale domains.	High internal reliability	Scale largely designed for use with migrant groups, and therefore does have a culturally and healthcare system specific element, which may limit its application
Earnshaw and Quinn, 2011 [52]	2	Instrument designed to assess stigma in healthcare on people living with any chronic illness. Items were organised into three domains examining internalised, enacted and anticipated stigma. Participants asked to indicate their opinion on a five‐point Likert scale on questions such as ‘It is my fault that I have a health condition’ and ‘I can't do a lot of things because I have a health condition’	Internalised/Enacted/Anticipated	People living with hepatitis HBV and HCV	Cronbach's alpha for the 12‐item scale was 0.93, indicating high reliability of the scale. Scale also had high construct validity.	Stigma theory underpinned scale design High internal reliability and construct validity	Initial design is for chronic illnesses generally and therefore may miss inter‐related social stigmas when applied to hepatitis
Social Impact Scale (Fife and Wright, 2000) [53]	2	Instrument measures four dimensions of stigma; social rejection, financial stigma, internalised stigma and social isolation. Participants divided into low and high stigma groups depending on number of ‘yes’ answers to questions such as ‘I feel I need to keep my illness a secret’. While a general instrument, it has been utilised in multiple instances in people living with hepatitis C	Internalised/Enacted	People living with hepatitis HBV and HCV	Cronbach's alpha for the subscales ranged between 0.85–0.90	Contains domains not usually included but that are important (e.g., financial instability)	Initial design for HIV and cancer and therefore may miss inter‐related social stigmas when applied to hepatitis Treatments are more developed since its design and therefore some measures may be outdated (e.g., changes in appearance)
Hepatitis B Quality Of Life Instrument (Spiegel et al., 2007) [54]	2	Primarily a tool to assess quality of life in people living with hepatitis B but includes a domain on stigma. Qualitative response‐based tool, with participants asked to ‘think aloud’ whereby they spoke their thoughts as they read items.	Internalised/Enacted	People living with hepatitis HBV	Cronbach's alpha for stigma domain specifically was 0.89 among 6 items, indicating high reliability of the scale	An overall quality of life scale with a specific stigma subscale, for an holistic look at health and wellbeing in relation to hepatitis B	Analyses using subscales may be less common and therefore less specific findings in relation to stigma

While the majority of studies in the sample referenced a specific tool used as the survey instrument, 19 (23%) of studies developed their own data collection tool, typically using existing literature to inform its development. In terms of type of stigma measured, 52 studies (64%) investigated multiple domains of stigma (50 (62%) internalised and enacted stigma, 2 (2%) internalised, enacted and anticipated). Enacted stigma was investigated in 70 studies (86%); internalised stigma in 62 (77%) and anticipated stigma in 2 studies (2%).

### 3.2 Synthesis of Study Findings

Findings from the included studies were synthesised to understand stigma experienced and expressed towards PLWVHB/C, and four distinct themes emerged: the health impact of stigma, the social impact of stigma, stigma in healthcare and reducing stigma (Table 2).

**TABLE 2:** Common themes from synthesising findings by study focus (people living with hepatitis B, people living with hepatitis C and people not living with hepatitis B or C).

	People living with hepatitis B	People living with hepatitis C	People not living with hepatitis B or C
Health impacts of stigma	Mixed evidence of disease progression and its association with stigma Stigma associated with anxiety and depression	Mixed evidence of disease progression and its association with stigma Stigma associated with depression	Stigmatising attitudes associated with reduced testing for hepatitis B and C Reduced stigma was associated with vaccination behaviour, but stigmatising attitudes were also present in those that had been vaccinated
Social impacts of stigma	Stigma associated with reduced quality of life Social isolation as a result of stigma	Stigma associated with reduced quality of life Social isolation as a result of stigma	Stigmatising attitudes towards PLWHB/C can be found in the workplace, and certain occupations may illicit more stigma (e.g., catering, childcare)
Stigma in healthcare	Too few studies among people living with hepatitis B and healthcare to draw conclusions	Disclosing status can result in discrimination in healthcare Stigma associated with reduced healthcare engagement	Most healthcare and dental workers willing to care for and work with PLWHB/C but some stigma exists Some evidence of discrimination (last appointment of the day/double‐gloving) and stigmatising attitudes found Self‐efficacy associated with more willingness to care for PLWHB/C
Reducing stigma	—	—	Increased knowledge mostly associated with reduced stigma. Where knowledge has not reduced stigma, it's suggested that not enough is done to address underlying fears Stigma associated with conservative beliefs Knowing someone with hepatitis associated with reduced stigma Videos of PLWHB/C aiming to raise awareness and testing can reduce stigma

#### 3.2.1 Health Impact of Stigma

Nine studies investigated disease progression in relation to stigma, and findings for both PLWHB and PLWHC were mixed. For PLWHB, three studies suggested there was no evidence between disease progression or time since diagnosis and stigma, while two studies suggested that those who are in an active phase of the virus experienced greater stigma. Similarly, three studies also suggested there was no evidence of disease progression in relation to stigma for PLWHC. Additionally, one study did find that an association between stigma and time since diagnosis was reduced when controlling for age. One study found a higher level of internalised stigma among PLWHC with advanced disease stage compared to PLWHB with early disease stage.

One study reported that self‐stigma was associated with reduced self‐management of hepatitis for PLWHB. For both PLWHB/C, stigma was associated with poorer mental health outcomes, most commonly depression (*n* = 3), followed by negative emotions (*n* = 2), and with one study investigating self‐esteem and one investigating anxiety. The role of intersectionality in the experience of stigma was not typically investigated by included studies, although one study found that among people living with HBV, those with a current psychiatric diagnosis experienced greater stigma compared to those without.

Among people not living with hepatitis, seven out of nine studies found a negative relationship between stigma and testing behaviours, whereby stigmatising attitudes were a barrier to testing. One study found reduced stigma was associated with greater intention to vaccinate, and two studies found that reduced stigma was associated with vaccination behaviour. However, three studies found that there was no difference in stigma between those vaccinated and those unvaccinated.

#### 3.2.2 Social Impact of Stigma

Stigma was associated with reduced quality of life, which included environmental and social life quality, for PLWHB/C (*n* = 2). For PLWHC, one study reported those who were RNA negative were more likely to report being left out of social situations than those who were RNA positive; similarly for PLWHB, one study reported stigma being associated with social isolation.

Among people not living with hepatitis B and C, two studies from China found around half of people were uncomfortable with PLWHB working in restaurants or childcare. Four studies investigated stigma in the workplace. In two studies from Japan, between a quarter and a third expressed stigmatising attitudes towards PLWHB/C. In one of these studies, they also found that a quarter would assume a person was a GBMSM, injected drugs, or had multiple sexual partners if they were living with hepatitis B or C, indicating a stigma around potential routes of acquisition. In a study from the USA, they found that knowing someone living with hepatitis C reduced the need for social distance from and increased willingness to provide services to someone living with hepatitis C. In Egypt, a study found more stigma directed to marketplace sellers living with hepatitis C if they were selling products like food, compared to if they were selling non‐perishables like clothing.

#### 3.2.3 Stigma in Healthcare

No studies included in the review investigated experiencing stigma in healthcare settings among PLWHB. For PLWHC, four studies investigated stigma in healthcare settings, three of which focused specifically on PWID. Findings in this population included reporting worse experiences at drug and alcohol services compared to people not living with HCV; experiencing stigma was associated with not undergoing liver disease staging and trying but being unable to access HCV treatment among women; and experiencing discrimination from healthcare workers was associated with reduced HCV treatment intention. Among PLWHC more generally, one study found that disclosure was associated with discrimination in healthcare settings.

There were 18 studies that recruited healthcare or dental workers and measured stigmatising attitudes. Due to the heterogeneity in the measures used to understand stigma among healthcare workers, it was difficult to synthesise the proportion expressing stigma, but generally, positive attitudes towards and a willingness to care for PLWHB/C were reported. However, stigmatising attitudes and practices were reported by some, such as having different clinical conducts (e.g., double gloving, additional infection control, giving PLWHB/C last appointment of the day), not accepting a colleague living with hepatitis B or C and a reluctance to offer testing due to fear of offending patients or their family. Two studies found that self‐efficacy was associated with more willingness to care for patients living with hepatitis B or C. Studies regarding healthcare and dental workers came from 11 different countries, and it seems likely that there would be regional variations regarding stigmatising attitudes and practices in healthcare settings.

#### 3.2.4 Reducing Stigma

Knowledge was most commonly researched in relation to stigmatising attitudes (*n* = 14), where most studies (*n* = 9) found that increases in knowledge were associated with reduced stigma. Where studies did not find an association, or in fact found a negative association between knowledge and stigma, it was suggested that the mechanisms of reducing stigma are complex. It was proposed that underlying fears may also need to be addressed so that increases in knowledge can reduce stigma. One study assessed the impact of a virtual seminar on HBV among medical students with the primary aim to increase knowledge, which found increased acceptability of a colleague living with hepatitis B, as well as reduced discomfort treating a patient living with hepatitis B.

Five studies found that either knowing someone or having contact with someone living with hepatitis reduced stigmatising attitudes. Less commonly researched were conservative attitudes and stigma (*n* = 3), but all studies found conservative attitudes were associated with increased stigma.

Two studies evaluated interventions aimed at reducing stigma towards individuals with HBV or HCV from members of the public. Online video interventions depicting affected population groups were found to be effective in reducing stigmatising attitudes among the general Australian public. An intervention in China which included crowdsourced images or videos to promote hepatitis testing found full exposure to the intervention was associated with significant stigma reduction.

### 3.3 Quality Assessment

Most cross‐sectional studies (*n* = 78) were of high quality (median AXIS score = 16, range 12 to 18). Despite the high quality of studies overall, one area that studies tended to do poorly on was the justification of sample size. Of the three non‐cross‐sectional studies evaluated using ROBINS‐I, one scored low, one scored moderate and one scored serious risk of bias. The study that was assigned serious was due to potential misclassification of intervention due to self‐reporting exposure status, which could be influenced by recall bias.

## 4 Discussion

This systematic review synthesised findings from 81 studies examining the measurement of, and factors associated with stigma towards and experienced by people living with HBV or HCV. The findings highlight the common nature of stigma experienced by this population across various global settings. Both internalised and enacted stigma were regularly reported, demonstrated through social avoidance, discrimination and negative attitudes from others upon disclosure of one's status. Fear of acquisition emerged as a predominant driver of stigmatising behaviours and attitudes.

The primary aim of this review was to understand the way in which stigma relating to PLWHB/C is measured and highlighted the wide range of tools and measures used to assess HBV/HCV‐related stigma. In line with HIV stigma research [19], some studies adapted existing scales developed for other disease contexts, while others created study‐specific questionnaires. However, the variability in measurement approaches may limit the comparability of findings across studies and contexts. Efforts to establish standardised, culturally validated instruments aligned with conceptual frameworks (e.g., The Health Stigma and Discrimination Framework [55]) could facilitate more robust and harmonised research in this area. Although the importance of adapting tools to the local context is evident, establishing a standardised framework or core set of measures could promote a harmonious balance between locally tailored instruments and comparability of data on an international scale. This is similar to a conclusion from a HIV stigma systematic review, suggesting that the people living with the condition would benefit from a more streamlined approach from the research field [19], although it is unclear currently how well this recommendation can be adapted in practice.

The World Hepatitis Alliance (WHA) has developed a survey designed to measure stigma experienced by people living with hepatitis B and C, in partnership with the European Centre for Disease Prevention and Control (ECDC), which was piloted in nine countries [56] The findings from this study should inform efforts to conceptualise a standardised framework, as should commonly used tools such as Brener & Von Hippel (2008) [32] and the 20‐item Toronto Chinese HBV Stigma Scale by Li et al. (2012) [23]. A more recent tool from Australia [57], which was utilised in one study in this review [41], compiled previous research into one question regarding experiences of stigma for the population of interest being studied to be used across MSM, PWID, PLWHB/C, people living with HIV and people who engage in sex work. The tool can be used for surveying the people in that community and broader public perceptions, and initial correlations with related tools (discrimination, internalised stigma, wellbeing) were high for HIV and PWID research, although less so for HCV [57]. It is yet to be seen how widely this single‐measure tool is to be adapted in research, but if reliability and validity can be confirmed, then a single measure tool could limit research burden on participants by reducing questionnaire length.

The main domains commonly assessed were internalised stigma (e.g., self‐blame, shame) and enacted stigma (e.g., experiences of discrimination), with less emphasis on anticipated stigma (e.g., expectations of discrimination). Effectively measuring anticipated stigma should not be overlooked in conceptual frameworks aiming to tackle hepatitis‐related stigma, given how it is used to understand avoidance of healthcare and social situations which can contribute to poorer quality of life. The experience of stigma was associated with numerous adverse psychosocial outcomes, including impaired quality of life, low self‐esteem, social isolation and depression. Notably, stigma also hindered timely diagnosis and treatment access, acting as a significant barrier to care. Internalised stigma and fears of discrimination often led individuals to conceal their status, delay testing and avoid disclosing to healthcare providers, impeding linkage to appropriate management and support services. Enacted stigma in healthcare settings, such as discrimination by providers or denial of services, further exacerbates barriers to accessing quality care. These findings underscore the detrimental impact of stigma on the well‐being and health outcomes of affected populations. As we push towards the WHO elimination goals, this emphasises the need for targeted interventions to address this issue at multiple levels to be integrated into elimination strategies.

Several factors were identified as contributing to higher levels of stigma, such as limited knowledge about HBV/HCV transmission and treatment, lower education levels and conservative beliefs. Conversely, increased familiarity and knowledge about HBV/HCV were associated with reduced stigmatising attitudes. These findings suggest that educational initiatives, like those used in Australia and China [25, 58], aimed at improving public understanding and dispelling misconceptions about HBV and HCV could be effective in mitigating stigma. However, it is crucial to note that such initiatives should be tailored to specific cultural and socioeconomic contexts to ensure their effectiveness and relevance. Additionally, where increased knowledge did not reduce stigmatising attitudes, it was suggested that interventions did not address people's underlying fears and concerns relating to hepatitis. Therefore, when developing interventions to reduce stigmatising attitudes, it is important that increases in knowledge are also addressing potential fear to maximise impact.

Furthermore, the review revealed that stigma towards individuals with HBV or HCV was prevalent not only among the general public, but also among healthcare professionals. This highlights the need for targeted training and awareness programmes within the healthcare sector to address stigmatising attitudes and ensure non‐discriminatory and compassionate care for affected individuals. All studies among healthcare and dental staff in this review (*n* = 18) were cross‐sectional, and it therefore appears there is a lack of published interventions for reducing stigma among healthcare workers. One study did look at the impact of an HBV seminar on reducing stigma among medical students, but its primary aim was around increasing knowledge; more interventions aimed at reducing stigma may be needed. Tools were often utilised for both PLWHB/C and people not living with hepatitis, which appeared to be consistent at measuring attitudes towards PLWHB/C, but it may be appropriate to have tools specific to the social setting being studied. For example, in healthcare settings, it may be more appropriate to know intent to discriminate against PLWHB/C and comfort treating PLWHB/C, as measured in Ishimaru et al. (2016), as opposed to generalised attitudes.

### 4.1 Limitations

While this review synthesised data from studies spanning multiple regions, the generalisability of findings may be limited by the geographical spread of the included studies. Geographic and cultural contexts can significantly limit the generalisability of findings related to hepatitis C stigma due to variations in cultural beliefs and norms [59, 60], economic factors and access to care [61], healthcare professional attitudes and service integration influenced by cultural contexts [36, 48], legal and policy frameworks across regions [62], as well as varying prevalence across the world. Thus, while there are commonalities, interpreting at the global level should be done with caution. It was, however, deemed important to look at the evidence internationally in this review to see what research has been conducted globally, given the aim to eliminate hepatitis as a public health threat by the year 2030.

Additionally, there was limited representation of Indigenous/First Nation people, whose experiences of stigma may differ due to distinct cultural contexts and marginalisation. Some studies that were culturally tailored to First Nation people were excluded during screening due to not meeting the quantitative inclusion criteria [63, 64]. As culturally tailored interventions may be more likely to be qualitative in nature, future reviews should include both qualitative and quantitative studies to ensure all groups of individuals who are disproportionately affected by HBV and HCV are included in research. There is also a need for larger studies for those groups who are disproportionately affected so that group‐level findings are not generalised to groups who may face different barriers and stigmatisation. The cross‐sectional nature of the evidence also precludes causal inferences about the relationship between stigma and its associated factors or outcomes. Future longitudinal and interventional studies, particularly among underrepresented populations, are warranted to establish temporal relationships and evaluate the effectiveness of stigma‐reduction strategies. Related to this, our ability to understand any temporal changes in stigma was limited due to the variation in countries included and the tools used to measure stigma.

Furthermore, the review aimed to focus specifically on stigma related to HBV and HCV; however, we should acknowledge interconnected stigmas associated with other characteristics such as homelessness, injecting drug use and migrant status. One of the more common measures of stigma (Brener & Von Hippel (2008)) found a high correlation between HCV‐related stigma and other potentially stigmatising conditions and populations [32]. Similarly, one study found a difference in the relationship between stigma and quality of life between HBV and HCV, suggesting that the different route of acquisition more commonly associated with each virus may be the cause. Disentangling stigma relating to the virus and to the method of acquisition appears to be difficult in research and may be true for the population being studied. Additionally, people may belong to multiple groups who face a disproportionally higher prevalence of HBV and/or HCV, and understanding the source of stigma in these instances may be even more difficult. Complementing quantitative studies with qualitative information should be used to aid in inferring causation around stigmatising experiences and understanding how PLWHB/C understand their intersecting group identities.

## 5 Conclusions

This systematic review underscores the pervasive nature of stigma experienced among PLWHB/C, and its detrimental impact on various aspects of their well‐being and health outcomes. In order to meet the WHO hepatitis elimination targets, the measurement of stigma should be considered an essential component in its own right, serving as evidence for the elimination of stigma and discrimination. Therefore, studies investigating stigma experienced by PLWHB/C should be conducted in countries where stigma has received little to no research to address this issue in the context of elimination targets. Understanding stigma as a driver of inequality is crucial, as it significantly impacts the hepatitis cascade of care and perpetuates health disparities. A standardised framework, such as those established in other disease contexts such as HIV, could be conceptualised to act as a starting point for the development of culturally validated local tools.

The findings highlight the need for targeted interventions addressing stigma, particularly through educational initiatives to improve knowledge and dispel misconceptions about hepatitis among the public, as well as healthcare practitioners where needed. Efforts to establish standardised measurement approaches and conduct longitudinal, interventional research in diverse global settings, including among underrepresented Indigenous/First Nations groups, are warranted to further advance understanding and develop effective strategies to combat HBV/HCV‐related stigma.

## Conflicts of Interest

The authors declare no conflicts of interest.

## Supporting information


**Data S1:** Data extraction.

**Data S2:** Summary of included papers.

## Data Availability

The data that support the findings of this study are available in the Supporting Information of this article.
