# Supplementary material for: Global Systematic Review of the Measurement of Stigma Associated With People Living With Hepatitis B or Hepatitis C Viruses
Source: J Viral Hepat. 2025 Aug 26;32(9):e70064. doi: 10.1111/jvh.70064 (PMC12379575; doi:10.1111/jvh.70064)
Supplement: Supplementary file 2 — Data S2: Summary of included papers. [file JVH-32-0-s001.docx]

**Summary of included papers**

| Citation | Country | Study design | Study population | Hepatitis focus | Sample size | Tool used to measure stigma | Type of stigma | Key stigma result |
| --- | --- | --- | --- | --- | --- | --- | --- | --- |
| Living with hepatitis |  |  |  |  |  |  |  |  |
| Abu Elhija et al. 2019 (28) | Israel | Cross-sectional | Patients | Both | 120 | Li et al, 2012 (20-item Toronto Chinese HBV Stigma Scale) | Internalised / Enacted | A significant association was found between the level of self-stigma and the stage of the disease at diagnosis. Higher levels of self-stigma were reported by Hepatitis C patients with advanced disease compared to Hepatitis B patients with early disease. No significant correlation was found between self-stigma and the lag-time from diagnosis to first visit to the liver clinic. |
| Ahmed et al. 2023 (93) | Pakistan | Cross-sectional | Patients | HBV | 432 | Earnshaw and Quinn, 2011 | Internalised | Self-stigma correlated with reduced quality of life and reduced self-management of hepatitis. |
| Balbinot et al. 2022 (94) | Brazil | Cross-sectional | Patients | HBV | 124 | Spiegel et al., 2007 (Hepatitis B Quality of Life Instrument) | Internalised / Enacted | Increased treatment time associated with increased stigma for those treated with tenofovir but not entecavir. |
| Brener et al. 2015 (33) | Australia | Cross-sectional | PWID | HCV | 416 | Brener & von Hippel, 2008 | Internalised / Enacted | HCW discrimination and self-stigma associated with reduced treatment intention but not discrimination from others. |
| Brener et al. 2015 (35) | Australia | Cross-sectional | First nation | HCV | 203 | Brener & von Hippel, 2008 | Internalised / Enacted | Perceived stigma associated with reduced quality of life but not experiences of discrimination. |
| Cabrera et al. 2015 (63) | Canada | Cross-sectional | Patients | HCV | 83 | Wright et al., (2007) | Internalised / Enacted | HCV stigma significantly associated with depression. |
| Drazic et al. 2013 (59) | Australia | Cross-sectional | Patients | Both | 77 | Fife and Wright, 2000 (Social Impact Scale) | Internalised / Enacted | Stigma associated with reduced HRQoL for PLWHC patients but not PLWHB. |
| Franklin et al. 2018 (71) | Zambia | Cross-sectional | Patients | HBV | 79 | Molina et al., 2013 (Stigma Scale for Chronic Illness) | Internalised / Enacted | Increased stigma associated with reduced disclosure to contacts. |
| Gupta et al. 2020 (27) | India | Cross-sectional | Patients | HBV | 150 | Li et al, 2012 (20-item Toronto Chinese HBV Stigma Scale) | Internalised / Enacted | Greater stigma associated with those with a current psychiatric diagnosis among people with PLWHB and not inactive carriers. |
| Han et al. 2018 (54) | China | Cross-sectional | Patients | HBV | 797 | Hepatitis B discrimination index modified from AIDS/STD Division of Policy & Sociology in the Joint United Nations Program on HIV/AIDS (UNAIDS) discrimination measure. | Enacted | Negative emotions (mental distress) associated with discrimination. |
| Higashi et al. 2020 (65) | USA | Cross-sectional | Patients | HCV | 159 | Berger et al., 2001 (HIV stigma scale) | Internalised / Enacted | Antibody reactive participants more likely to report being left out of social situations than RNA reactive. |
| Hopwood et al. 2010 (55) | Australia | Cross-sectional | Patients | HCV | 504 | Tool developed by authors. Questions not stated | Enacted | Being refused medical treatment because of HCV had disclosed more widely. |
| Huang et al. 2016 (30) | China | Cross-sectional | Patients | HBV | 1,236 | Li et al, 2012 (20-item Toronto Chinese HBV Stigma Scale) and Cotler et al. (2012) | Internalised / Anticipated / Enacted | Controls expressed discomfort with close contact (45%) or sharing meals with PLWHB patients (39%) and believed PLWHB patients should not be allowed to work in restaurants (58%) or childcare (44%). having a family member with PLWHB or having been tested for HBV or vaccinated against HBV did not significantly impact stigma. |
| Jones et al. 2022 (66) | USA | Cross-sectional | PWID | HCV | 269 | Latkin et al. 2013 | Internalised / Enacted | Stigma associated with not undergoing liver disease staging and attempting but unable to access HCV treatment among women. |
| Le et al. 2019 (95) | Vietnam | Cross-sectional | Patients | HBV | 298 | Luoma et al., 2013 | Internalised / Enacted | Two comorbidities associated with Blame/Judgement, 1 or 2 comorbidities associated with reduced feeling discriminated against. Comorbidities not associated with shame. |
| Li et al. 2020 (31) | China | Cross-sectional | Patients | HBV | 401 | Li et al., 2012 (20-item Toronto Chinese HBV Stigma Scale) | Internalised / Enacted | Stigma associated with reduced physical, psychological, social and environmental QoL. |
| Medvinsky et al. 2014 (62) | USA | Cross-sectional | Patients | HCV | 126 | Fife and Wright, 2000 (Social Impact Scale) | Internalised / Enacted | Stigma correlated with depression; stigma did not vary by treatment status. |
| Mohamed et al. 2012 (96) | Malaysia | Cross-sectional | Patients | HBV | 483 | Tool developed by authors and assessed whether participants were (1) Worried of spreading HBV to family and friends (2) worried ever since diagnosis | Internalised | Receiving treatment associated with less worry since diagnosis, but not worry about transmission to family, presence of cirrhosis not associated with worry since diagnosis/family transmission. |
| Noor et al. 2016 (60) | Pakistan | Cross-sectional | Workplace | HCV | 228 | Earnshaw and Quinn, 2011 | Internalised | Internalised stigma correlated with reduced self-esteem. |
| Poorkaveh et al. 2012 (64) | Iran | Cross-sectional | Patients | HBV | 320 | Spiegel et al., 2007 (Hepatitis B Quality Of Life Instrument) | Internalised / Enacted | More stigma associated with past diagnosis compared to recent, stigma correlated with anxiety and depression. |
| Rafique et al. 2014 (58) | Pakistan | Cross-sectional | Patients | Both | 140 | Tool not stated/devised by authors | Internalised / Enacted | Experience of stigma did not differ between duration of illness and whether someone was living with either HBV/HCV/both. |
| Rashidi et al. 2020 (34) | Australia | Cross-sectional | First nation | HCV | 123 | Brener & von Hippel, 2008 | Internalised / Enacted | HCV stigma associated with reduced intention to treat. |
| Saine et al. 2020 (97) | USA | Cross-sectional | Patients | HCV | 265 | Saine et al, 2020 (HCV Stigma Scale (HCV-SS) Score (modified from Berger et al, 2001)) | Internalised / Anticipated / Enacted | No difference in stigma between people living with HCV and people living with HCV and HIV. Previously treated but not cured associated with greater stigma among those living with HCV/HIV but not those just living with HCV. |
| Soltan et al. 2018 (75) | Egypt | Cross-sectional | Patients | HCV | 260 | Cabrera et al, 2014 (Hepatitis C Stigma Scale) | Internalised / Enacted | Duration of HCV not related to stigma when controlling for age, but significant in univariable. |
| Yilmaz et al. 2022 (72) | Turkey | Cross-sectional | Patients | HBV | 505 | Tool developed by authors, including questions such as "Has your illness negatively affected your relationship with your parents?" | Enacted | Experiencing stigma was associated with disclosure. |
| Yousaf et al. 2021 (61) | Pakistan | Cross-sectional | Patients | Both | 120 | Cotler et al., 2012 | Internalised / Enacted | Stigma correlated with depression and social isolation. |
| Yozgat et al. 2021 (98) | Turkey | Cross-sectional | Patients | Both | 166 | Holzemer et al., 2007 (Berger stigma scale) | Internalised / Enacted | Stigma did not differ by disease stage for hepatitis B and C. |
| Yu et al. 2015 (99) | China | Cross-sectional | Patients | HBV | 797 | Hepatitis B discrimination index modified from AIDS/STD Division of Policy & Sociology in the Joint United Nations Program on HIV/AIDS (UNAIDS) discrimination measure. | Internalised | Stigma was strongly associated with negative emotions. |
| Zhu et al. 2019 (74) | China | Cross-sectional | Patients | HBV | 578 | CHBQOL generated from SF-36 HRQOL | Enacted | No difference in social stigma among HBV DNA levels (>=2000 iU/ml vs. <2000) but more social stigma associated with abnormal ALT scores and HBeAg positivity. |
| Not living with hepatitis |  |  |  |  |  |  |  |  |
| Adjei et al. 2022 (26) | Ghana | Cross-sectional | Public | HBV | 971 | Li et al, 2012 (20-item Toronto Chinese HBV Stigma Scale) | Internalised / Enacted | Stigmatising attitude associated with reduced HBV testing. |
| Akazong et al. 2020 (100) | Cameroon | Cross-sectional | Healthcare workers | HBV | 398 | Not stated/devised by authors | Internalised | 68% had a positive attitude towards people living with HBV. |
| Brener et al. 2008 (32) | Australia | Cross-sectional | University | HCV | 110 | Shrum, Turner, and Bruce, 1989 | Internalised / Enacted | University students: HCV stigmatising attitudes associated with religious fundamentalism and conservatism and attitudes towards PWID; HCWs: HCV stigmatising attitudes associated with negative attitudes towards PWID. |
| Brener et al. 2013 (39) | Australia | Cross-sectional | MSM | HCV | 590 | Brener & von Hippel, 2008 | Internalised / Enacted | Positive attitudes to person with HCV associated with ever having an HCV test. |
| Brener et al. 2014 (40) | Australia | Cross-sectional | Mental Health | HCV | 117 | Brener & von Hippel, 2008 (adapted from AIDS Attitude Scale) | Internalised / Enacted | Attitude towards people living with HCV correlated with comfort in clients home and capable of working with client, but not when controlling for attitude towards PWID. |
| Brener et al. 2018 (80) | Australia | Cross-sectional | Healthcare workers | HCV | 90 | Tool developed by authors. (1) How supporting would you be of each client to undertake HCV treatment? | Enacted | Negative attitude towards PWID was associated with reduced support for HCV treatment and support higher when person was not injecting. |
| Brener et al. 2022 (37) | Australia | Cross-sectional | Healthcare workers | HBV | 551 HCWs & 199 Students | Brener & von Hippel, 2008 | Internalised / Enacted | Stigma levels were low, no difference in stigma measures between HCW and students except more HCW agreed people with HBV got what they deserved. |
| Broady et al. 2020 (41) | Australia | Cross-sectional | Public | HCV | 1,001 | Brener & von Hippel, 2008 | Internalised / Enacted | Voting for conservative parties in the last election associated with more stigmatising HCV attitudes. Knowing someone with HCV associated with less stigmatising attitude. |
| Broady et al. 2021 (81) | Australia | Cohort | Public | Both | 2,010 | Broady et al., 2018 | Internalised / Enacted | Short videos of lived experience improved attitudes, controllability (condition under the person's control), personal distance and opinions on how individuals should be treated in health and social settings for HBV and HCV; progressive political beliefs (vs. conservative) associated with improved attitudes, controllability, personal distance and opinions. |
| Cama et al. 2021 (36) | Australia | Cross-sectional | Healthcare workers | HBV | 551 | Brener & von Hippel, 2008 | Internalised / Enacted | HBV stigmatising attitudes associated with having concerns around providing care to PLWHB, knowing someone with HBV associated with reduced concerns. |
| Chen et al. 2020 (52) | China | Cross-sectional | Antenatal | HBV | 270 | Feng et al., 2011 (Chronic Hepatitis B Virus Infectors' Discrimination Measurement Scale) | Internalised / Enacted | Greater stigma correlated with worse mental and physical health. |
| Dam et al. 2016 (76) | Vietnam/USA | Cross-sectional | Migrants | HBV | 842 from Vietnam & 170 from USA | Cotler et al., 2012 | Internalised / Enacted | Not being previously tested and unwilling to test was associated with increased stigma. Knowledge was slightly correlated with stigma. |
| Eguchi et al. 2013 (53) | Japan | Cross-sectional | Public | Both | 3,129 | Adapted from Balfour et al., 2009 | Internalised / Enacted | 36% worry about transmission with colleague with HCV, 32% would avoid contact, 24% would have prejudiced opinions; Increased knowledge was associated with reduced worry, avoidance and prejudice. |
| Eguchi et al. 2014 (78) | Japan | Cross-sectional | Workplace | Both | 3,129 | Tool developed by authors, including question, "If I found that people with whom I work were infected with HIV or HBV/HCV, I think I would look at him/her to be a homosexual, someone who engaged in sexual relationships with an unspecified number of people, or a drug addict" | Enacted | 24% expressed prejudice towards colleague with HBV/HCV. Increased hepatitis knowledge associated with reduced prejudice towards colleague with HBV/HCV. |
| Frazer et al. 2011 (48) | Ireland | Cross-sectional | Healthcare workers | HCV | 560 | Richmond et al., 2007 | Internalised / Enacted | 94% were willing to treat patients who acquired HCV, 52% would use additional protective measures. Increased knowledge score associated with more positive attitudes. |
| Ghazzawi et al. 2023 (77) | Sierra Leone | Cross-sectional | Public | HBV | 306 | Tool developed by authors, including - (1) Would you have concerns sharing food or utensils with someone who has Hepatitis B? (2) Would you have concerns having casual contact or working with someone who has Hepatitis B? | Enacted | High knowledge of HBV associated with greater stigma, no difference in stigma between vaccinated and unvaccinated and HBV positive/not. |
| Hamdiui et al. 2018 (101) | Netherlands | Cross-sectional | Migrants | HBV | 379 | van der Veen et al., 2014 | Internalised | Shame and stigma predictors of reduced intention to test for HBV both when cost is and isn't involved. |
| Hang Pham et al. 2019 (82) | Vietnam | Cross-sectional | Healthcare workers | HBV | 314 | Adapted from Chao et al., 2010 & Wang et al., 2016 | Internalised | 27% would have concern working with someone who is a PLWHB and 21% would have concern if child was in a class with classmate who is a PLWHB. |
| Hurt et al. 2020 (51) | USA | Cross-sectional | Healthcare workers | HCV | 76 | Richmond et al., 2007 | Internalised / Enacted | Scores reflect compassion towards PLWHC and willingness to provide care. |
| Ishimaru et al. 2016 (42) | Japan | Cross-sectional | Healthcare workers | Both | 992 | Tool developed by authors, including - (1) Avoiding contact with an HBV/HCV infected colleague, (2) anxiety regarding the potential risk of infection from an HBV/HCV-infected colleague, and (3) expressing views such as that an HBV/HCV-infected colleague may be homosexual, have multiple sexual partners, or be a drug user. | Internalised / Enacted | Exposure to hepatitis, previous contact with colleague with hepatitis and increased knowledge of hepatitis associated with acceptance of colleague with hepatitis. Avoiding contact with colleague with hepatitis associated with reduced willingness to accept. No difference by vaccination status. |
| Ishimaru et al. 2017 (43) | Vietnam | Cross-sectional | Healthcare workers | Both | 400 | Ishimaru et al., 2016 | Internalised / Enacted | 87% accepted colleagues contacting patients as long as low viral levels and not performing high risk procedures; agreement that colleagues with HBV/HCV should disclose status and caring for a patient with HBV/HCV in the past year was associated with acceptance of colleague with HBV/HCV. |
| Ishimaru et al. 2017 (44) | Vietnam | Cross-sectional | Healthcare workers | Both | 400 | Ishimaru et al., 2016 | Internalised / Enacted | 73% willing to care for HBV/HCV patients, 9% avoid going near patients with HBV/HCV. Self-efficacy protects oneself was associated with willingness to care, stigmatising attitude associated with reduced willingness to care. |
| Ishimaru et al. 2018 (45) | Thailand | Cross-sectional | Healthcare workers | HCV | 546 | Ishimaru et al., 2016 | Internalised / Enacted | Fear of transmission associated with reduced willingness to care for patient with HCV; self-efficacy around protecting self from infection associated with increased willingness to care. |
| Jin et al. 2022 (102) | Australia | Cross-sectional | Migrants | HBV | 396 | Not stated/devised by authors | Enacted | Stigmatising attitude towards PLHBV associated low knowledge. |
| Korkmaz et al. 2016 (49) | Iran | Cross-sectional | Healthcare workers | HCV | 239 | Richmond et al., 2007 | Internalised / Enacted | 67% displayed negative attitudes; lack of knowledge was associated with more negative attitudes; 74% double gloved, 49% gave HCV patients last appointment of the day. |
| Leng et al. 2016 (69) | China | Cross-sectional | Migrants | HBV | 903 | Tool developed by authors. (1) Are you willing to accept gifts from hepatitis B patients or carriers? (2) Are you willing to shake hands with or hug them? (3) Are you willing to have dinner with them? (4) Do you think parents should let their children play with hepatitis B-infected children? (5) Do you think parents should accept their child marrying a hepatitis B-infected person? | Enacted | Increased knowledge associated with reduced discrimination; fear of HBV and receiving vaccination associated with discrimination |
| Li et al. 2012 (23) | Canada | Cross-sectional | Migrants | HBV | 343 | Li et al, 2012 (20-item Toronto Chinese HBV Stigma Scale) | Internalised / Enacted | Stigma associated with reduced HBV testing. |
| Li et al. 2021 (103) | China | Cross-sectional | Public | HBV | 22,618 | Leng et al., 2015 | Enacted | The level of PLWHB discrimination in the central and southern regions was generally low, and the level of PLWHB discrimination in the eastern coastal regions was higher. |
| Marley et al. 2022 (29) | China | Cross-sectional | Primary care patients | Both | 750 | Li et al, 2012 (20-item Toronto Chinese HBV Stigma Scale) | Internalised / Enacted | Testing associated with higher stigma. |
| Maxwell et al. 2012 (104) | USA | Cross-sectional | Public | HBV | 1,735 | Tool developed by authors, including "Doctors recommendation for people to avoid people with Hep B" | Enacted | Those with higher knowledge expressed more stigma. |
| Mohamad et al. 2016 (105) | Egypt | Cross-sectional | Workplace | HCV | 300 | Tool developed by authors developed using methodology from Hinkin, 1998 | Internalised / Enacted | More avoidance, pity and stigma were displayed in a perceived higher contagion risk situation (seller of bread) as opposed to low risk (seller of shoes). |
| Park et al. 2023 (106) | USA | Cross-sectional | Healthcare workers | HCV | 96 | Not stated/devised by authors | Not stated | If patient expressed stigma and a lack of knowledge of HCV more likely to prescribe treatment. |
| Perumalswami et al. 2023 (107) | USA | Cross-sectional | Migrants | HBV | 162 | Maxwell et al., 2010 | Internalised / Enacted | Stigma associated with reduced screening self-efficacy and reduced screening behaviour. |
| Rose et al. 2013 (38) | Australia | Cross-sectional | Mental Health workers | HCV | 117 | Brener & von Hippel, 2008 | Internalised / Enacted | 81% believe no one deserves to have HCV; 19% believed people who inject drugs are to blame for spread of HCV in Australia; More positive attitudes to PWID were correlated with more positive attitudes to people living with HCV. |
| Sasaki et al. 2014 (73) | Japan | Cross-sectional | Workplace | Both | 3,129 | Tool developed by authors. (1) Would you be anxious about possibly becoming infected from a co-worker who carries the hepatitis virus? (2) Would you avoid contact with a co-worker who is infected with a hepatitis virus as far as possible? (3) Would you be biased against a co-worker who is infected with a hepatitis virus, suspecting that the co-worker may be a homosexual or a drug user or that they have multiple sexual partners?” | Internalised / Enacted | 36% anxious about becoming infected from a co-worker who carries hepatitis, 32% would avoid contact as far as possible, 24% would be bias towards co-worker (assuming they were homosexual/drug user/multiple sexual partners). |
| Shen et al. 2020 (25) | China | Retrospective cohort | MSM | HBV | 470 | Li et al, 2012 (20-item Toronto Chinese HBV Stigma Scale) | Internalised / Enacted | Exposure to crowdsourced intervention reduced stigmatising attitudes 4 weeks afterwards. |
| Souza et al. 2017 (50) | Brazil | Cross-sectional | Dental workers | HCV | 306 | Richmond et al., 2007 | Internalised / Enacted | 98% reported positive attitudes towards people living with HCV; 95% willing to treat; 64% used additional infection control procedures and 40% double gloved; no correlation between knowledge and attitudes. |
| Srinivasan et al. 2022 (79) | India | Cross-sectional | Dental workers | HBV | 84 | Jain et al., 2014 | Enacted | 35% would not work in the same environment as someone with HBV, 94% believe dentists are ethically obligated to treat someone with HBV and 58% believed they should be treated in a different clinic. Knowledge of HBV had no effect on stigmatising attitude. |
| Suarez et al. 2014 (67) | USA | Cross-sectional | Workplace | HCV | 164 | Adapted from Herek & Capitanio, 1993 and Wark & Galliher, 2007 | Enacted | Having a friend or acquaintance reduced need for social distance from PLWHC and increased comfort providing services. |
| Tan et al. 2020 (108) | Singapore | Cross-sectional | Public | HBV | 784 | Adapted from Holzemer et al., 2007 & Kalichman et al., 2005 | Internalised / Enacted | Reduced stigma associated with screening and vaccination. |
| Todd et al. 2008 (68) | Afghanistan | Cross-sectional | Healthcare workers | HBV | 114 | Not stated/devised by authors | Internalised | 8% reported fear of offending patient or family as a reason to not test. |
| Tu et al. 2022 (109) | China | Cross-sectional | University | HBV | 5,130 | Tool adapted from "Propagandist Education Points of Knowledge for Hepatitis B Prevention and Control" and "Guidelines for the Chronic Hepatitis B Prevention and Control 2019" | Enacted | HBV knowledge associated with more positive attitudes to people living with HBV, testing and vaccination behaviours. |
| Van der Veen et al. 2014 (110) | Netherlands | Cross-sectional | Migrants | HBV | 355 | Cunningham et al., 2002 | Internalised / Enacted | Low intention to test associated with higher HBV stigma and shame. |
| Wada et al. 2016 (46) | Japan | Cross-sectional | Healthcare workers | Both | 992 | Ishimaru et al., 2016 | Internalised / Enacted | Anxiety around infection risk and prejudice associated with reluctance to care. Self-efficacy and previously treating patient with hep in past year associated with increased willingness to care. |
| Wakayama et al. 2021 (56) | Brazil | Cross-sectional | Dental workers | HBV | 550 | Tool developed by authors, including questions such as "Would you receive care from a dentist with hepatitis B?" | Enacted | 58% wouldn't receive care from a dentist with HBV; 54% wouldn't hire an assistant with HBV; 40% have different clinical conducts for patients with HBV. |
| Wang et al. 2009 (57) | Taiwan | Cross-sectional | University | HBV | 328 | Wang et al, 2006 | Internalised | Over three quarters wouldn't tell friends if they were a carrier and 84% would be afraid of getting HBV from friend even when immune. |
| Yang et al. 2023 (83) | USA | Cohort | University | HBV | 40 | Adapted from Al-Hazmi et al., 2015 and Al Wutayd et al., 2019 | Enacted | Virtual seminars increased acceptance of a colleague with HBV, reduced concerns about shaking hands or hugging person with HBV, reduced uncomfortableness caring for a patient with HBV. |
| Yu et al. 2016 (99) | China | Cross-sectional | Public | HBV | 6,538 | L. J. Yu et al., 2016 | Enacted | Vaccination was associated with less HBV discrimination; knowledge was not associated but fear was significantly associated with discrimination. |
| Both |  |  |  |  |  |  |  |  |
| Brener et al. 2009 (111) | Australia | Cross-sectional | D&A services | HCV | 240 | Not stated/devised by authors | Enacted | HCV positive reported worse experiences on the most part compared to HCV negative. |
| Cheng et al. 2017 (24) | USA | Cross-sectional | Public | HBV | 404 | Li et al, 2012 (20-item Toronto Chinese HBV Stigma Scale) | Internalised / Enacted | Stigma not related to knowledge, more recently visited primary care associated lower stigma, stigma not related to screening. |
| Cotler et al. 2012 (5) | USA | Cross-sectional | Migrants | HCV | 201 | Adapted from Holzemer et al, 2007 & Kalichman et al, 2005 | Internalised / Enacted | Increases in knowledge correlated with reduced stigma towards PLWHBV. |
| Huang et al. 2016 (112) | China | Cross-sectional | Public | HBV | 1,236 | Survey developed by authors. Questions not stated | Internalised | Roughly half controls said PLWHB should not be allowed to work in restaurants or work with children. |
